# Cold-Storage Problems

**Published:** 1911-04-22

**Authors:** 


### Hospital Architecture and Construction.

[Communications on this subject should be marked "Architecture" in the left-hand top corner of the envelope.]

### COLD-STORAGE PROBLEMS.

### II.—METHODS OF MECHANICAL REFRIGERATION.

There are three practical methods of producing temperatures desired in cooling rooms, boxes, or tanks on the compression system, namely, (1) direct expansion, (2) brine circulating, and (3) air circulating systems.

In the first the liquid ammonia is allowed to expand in pipes placed in the rooms to be cooled and in process of expansion absorbs the heat from the atmosphere. In the second, instead of being exposed to the atmosphere, the expansion coils are placed in an insulated tank containing brine; the expansion lowers the temperature of the salt-impregnated water, which remains in liquid form at temperatures far below the freezing point of pure water. This brine is then pumped through coils in the rooms to be cooled and returns to the tank to be used again. In the third method (namely air circulation) expansion coils are placed in an insulated box or chamber, through which the air is drawn to be cooled by passing over coils and distributed to the cooling rooms by fans, being abstracted therefrom by ducts to the pipe room to repeat the circuit again.

#### The Differential Values of the Three Systems.

These three systems, the direct expansion, the brine, and the air circulating, are each specially adapted for different purposes. The direct expansion is most commonly used, as it can be installed at less expense than the others. The brine system, although more costly, is sometimes more advantageous. For instance, when it is desired to run the compressor only a part of the day the brine pump only is kept running to circulate the cooling fluid. Also, when there are many small boxes or chambers to be cooled, it is more difficult to control the temperatures with a small expansion valve, but with the constant circulation of the brine the temperatures can be perfectly regulated. The air circulation system is used in the majority of abattoir cold storage, meat chilling, and freezing plants.

Refrigerating capacity is measured on the basis of the cooling effect of melting one ton of ice; a 10-ton refrigerating machine, when operated continuously for 24 hours, does under certain conditions the same amount of cooling as would be produced by melting ten tons of ice. In selecting the size of machine required for any plant, it is necessary to get one large enough to do the work when the maximum cooling is demanded; for this reason it is best to have a machine much larger than the average consumption of ice.

#### Ice-making Plant.

For cold-storage purposes or for work in which the demand upon the machine is constant during the entire 24 hours a 3-ton machine will, under certain conditions, take the place of 3 tons of ice, but if 3 tons of ice are needed in 6 hours it would require a machine four times as large (or 12 tons). It is, of course, possible to conserve the whole or portions of the cooling energy for use when required. Ice-making may generally be classified into two systems, namely, the "can" system and the drum system. The former is more extensively used, and is carried out by suspending a number of cans which taper slightly and are filled with water in salt brine to within 2 or 3 inches of their tops. The brine is kept at a temperature of 16° to 20° F. by means of refrigerating coils and is circulated round the cans till the water becomes solid ice. The cans are then removed and immersed in warm water for a few minutes to loosen the ice so that it may be tipped out. The "can" system of ice-making is the least expensive in first cost and also the most economical to work. The ice will normally be opaque, but certain improvements have made it possible to make the ice clear if so desired. The drum system is operated as follows: In a wooden tank are placed a series of hollow galvanised iron drums or cells from 10 to 14 inches apart, through which cold brine is circulated. The space between the series of cells is filled with water to be frozen, which, during freezing, is agitated by being made to rise and fall gently. Ice forms on the sides of the drums, gradually increasing in thickness until the two plates of ice formed on opposite drums join in the centre to make a single block. Warm brine is then circulated through the drums to loosen the blocks, which are lifted out of the tank by means of hooks or ropes frozen into them.

Cell-ice is made in blocks weighing from 4 to 6 cwt. and from 8 to 14 inches in thickness, which are of convenient size for handling.

#### Refrigerating Machinery.

The same machine is used to produce ice for refrigerating purposes, but it must be borne in mind that a 5-ton refrigerating machine will not produce 5 tons of ice. Refrigerating machines are rated in tons refrigerating capacity, and by a ton is meant, as before stated, the cooling effect equivalent to the melting of a ton of ice in 24 hours. Further, the ice-making capacity is less than the refrigerating capacity, as the water must be cooled from its natural temperature to 32° F. before freezing commences; in addition to this the freezing cans must be refrigerated, which requires energy equal to cooling a box of that size to 32° F. In consequence of these antagonistic properties the ice-making capacity is only from one-half to two-thirds of the refrigerating capacity.

When new buildings have to be provided they should as far as possible be of cubical shape, so that the outer walls offer the minimum amount of surface to the atmosphere combined with the maximum storage capacity. The machinery and the boilers may be placed under a separate roof, but adjoining, and the boilers, if steam-power is used, should be as far as possible from the cold chamber—seven to eight feet is generally found to be the most useful height for cold rooms, and it is desirable to have walls built with an air-space in their thickness, to form a non-conductive area.

Insulation is the keynote to an efficient service of cold storage, as by it fuel is economised, and if the scheme of insulation were perfect the plant would have nothing to do after lowering the temperature of the chamber but keep down the difference caused by heat from food stored and the slight loss from leakages at doors, etc., and the most popular insulating materials are: first, silicate cotton, free from slag globules; second, granulated cork $\frac{1}{16}$ to $\frac{3}{16}$ of an inch grains, free from dust and outside bark; and, third, flake charcoal. The required thicknesses for equivalent efficiency are respectively 7, 8, and 9 inches. The cork is half the weight of either of the others and costs £9 10s. per ton, whereas cotton and charcoal cost £5 to £6 10s. per ton.

A type of modern insulation might have inside the brick hollow wall 5 inches of silicate cotton, then $\frac{7}{8}$-inch thick grooved and tongued matchboarding, then a layer of $1\frac{1}{2}$ inch granulated cork slabs finished with a hard cement face well-trowelled. The ceiling might be similarly treated and the floor would be of asphalte turned up at the sides. Some authorities claim that cement is not desirable as a wall-finish, as it has been supposed that schizomycetes settle on such walls and create a strong mouldy smell. This, however, it not altogether proved by experience, and it would seem that supersaturation had been greatly in evidence to develop such growths. It is important to have double doors, on either side of a lobby if possible, at the entrance, so as to maintain as far as practicable an air-lock between the extreme temperatures of the cold chamber and the external atmosphere.

The method of illuminating the interior of a store is an important matter, both as regards heat and smell, and the most desirable form is electricity, as will be readily seen from the following table.

$5\frac{1}{2}$ cubic feet gas consumption gave about 900 units of heat.
One composite candle gave about 110 units of heat.
One 16 c.p. lamp gave about 30 to 40 units of heat.

The question, "What does artificial refrigeration cost?" is rather a difficult one to answer, for there are so many factors to be considered in each individual case.

For, instance, a modern plant (exclusive of motive power, insulation, and lagging) of 500 C.F. power would cost £110, whereas one of double the capacity, 1000 C.F. power, would cost £118. The writer has in mind a case of a cold store 15 feet 6 inches by 15 feet 6 inches by 7 feet high where the cost worked out to 5s. 9d. per cubic foot complete, including plant, motive power, insulation and necessary pipe lagging.

It may be said, however, that the working cost is practically that of the power required to run the compressor, and this is very slight. The machine is generally run only when the rest of the machinery is running, and therefore requires but little time and attention.

A small installation of 400 C.F. capacity cost £333 complete, including refrigerating plant, motive power, ice tank, larder cabinet, insulation, etc. A small installation to make ice and cool a room, completely erected, including all necessary plant, motive power, and insulation, would cost about £250.

